# Understanding the ecology and evolution of host–parasite interactions across scales

**DOI:** 10.1111/eva.12294

**Published:** 2015-08-20

**Authors:** Rachel M. Penczykowski, Anna‐Liisa Laine, Britt Koskella

**Affiliations:** ^1^ Department of BiosciencesMetapopulation Research CentreUniversity of HelsinkiHelsinkiFinland; ^2^ BiosciencesUniversity of ExeterTremoughUK; ^3^ Integrative BiologyUniversity of CaliforniaBerkeleyUSA

**Keywords:** coevolution, eco‐evolutionary dynamics, host–parasite, local adaptation, parasite‐driven evolution, spatial structure, spatiotemporal, time shift

## Abstract

Predicting the emergence, spread and evolution of parasites within and among host populations requires insight to both the spatial and temporal scales of adaptation, including an understanding of within‐host up through community‐level dynamics. Although there are very few pathosystems for which such extensive data exist, there has been a recent push to integrate studies performed over multiple scales or to simultaneously test for dynamics occurring across scales. Drawing on examples from the literature, with primary emphasis on three diverse host–parasite case studies, we first examine current understanding of the spatial structure of host and parasite populations, including patterns of local adaptation and spatial variation in host resistance and parasite infectivity. We then explore the ways to measure temporal variation and dynamics in host–parasite interactions and discuss the need to examine change over both ecological and evolutionary timescales. Finally, we highlight new approaches and syntheses that allow for simultaneous analysis of dynamics across scales. We argue that there is great value in examining interplay among scales in studies of host–parasite interactions.

## Introduction

**Figure 1:**
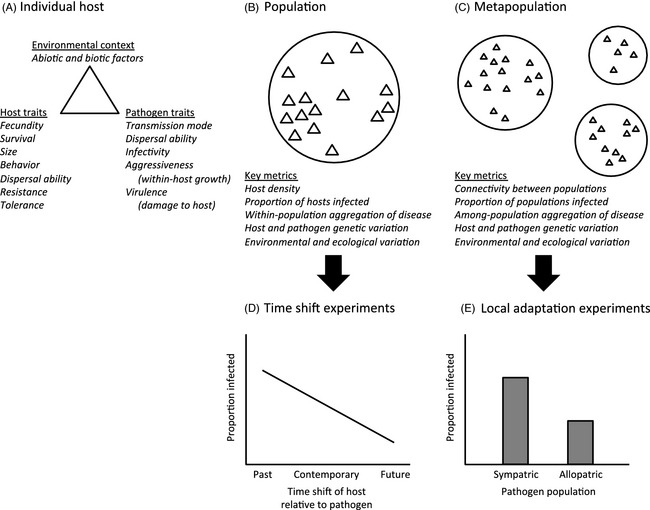
Schematic of hierarchical spatial scales of host–parasite interactions (A–C), and expected results from tests of parasite adaptation measured over time (D) or space (E). (A) The risk and consequences of infection for an individual host depend on the interaction between host traits, pathogen traits, and the surrounding abiotic and biotic environment. (B) The prevalence and spatial distribution of disease in a population, and ecological and evolutionary consequences of infection, are shaped by variation in host traits, pathogen traits and environmental factors over small spatial scales. (C) Within‐host and among‐host processes interact with larger‐scale environmental variation to determine the prevalence and spatial structure of disease at the metapopulation level. Cartoon representations of results of from (D) a time shift experiment in a single population, where the pathogen is most infective to hosts from the past and maladapted to hosts from the future and (E) a local adaptation experiment in a metapopulation, in which the pathogen is locally adapted to sympatric host populations.

Spatiotemporal variation in disease occurrence generates variation in the intensity of selection on hosts and parasites, which in turn shapes occurrence patterns. Studying patterns of disease prevalence at different spatial and temporal scales therefore offers a glimpse into both the potential for and result of (co)evolution of hosts and their parasites. Key insight into epidemiological and evolutionary processes can be gained by studying host–parasite interactions at spatial scales ranging from individuals to entire continents and at temporal scales ranging from within an individual's lifespan to thousands of generations. These scales are inherently hierarchical, as within‐host processes at the smallest spatial scales underlie among‐host processes in populations, and groups of populations interact with each other in metapopulations (Fig. 1A–C). Temporal scales are similarly nested, as parasite dynamics within an individual host's lifespan shape disease dynamics during epidemics, which in turn drive disease occurrence patterns and selection pressures over longer coevolutionary timescales. At each scale, the observed disease outcome arises from the interaction of the host, parasite, and surrounding abiotic and biotic environment (Laine 2008; Wolinska and King 2009; Duffy et al. 2012). In this review, we begin by describing spatiotemporal variation in disease occurrence patterns. We then examine what we have learned about host–parasite interactions across scales independently, including the use of local adaptation studies and time shift experiments to gain information on the spatial and temporal scales of coevolution as well as the specificity of the interaction. Finally, we emphasize the novel insights that can be gained through the combination of data sets from across scales and highlight new approaches that have examined multiple scales simultaneously. Throughout, we focus on three case studies involving diverse taxa and habitats (a plant–powdery mildew interaction in meadows, zooplankton–yeast in lakes and bacteria–phage from tree leaves; Table 1) to illustrate both the types of approaches that can be used and the general insights that can be gained through the study of hosts and parasites across scales. These study systems represent our respective areas of expertise, but are also different enough to allow some assessment of the generality of the phenomena discussed.

**Table 1:** Key features of the three model host–pathogen systems discussed throughout this review

	*Plantago lanceolata–Podosphaera plantaginis* (plant–powdery mildew)	*Daphnia dentifera–Metschnikowia bicuspidata* (zooplankton–yeast)	Bacteria–phage from horse chestnut trees (*Aesculus hippocastanum*)
Host			
Size (longest axis)	10–20 cm	1.5 mm	0.5–5 μm
Lifespan	Perennial, up to 7 years	Up to 2 months	Unknown
Reproduction	Sexual (outcrossing) and asexual (side rosettes)	Cyclically parthenogenetic (sexual resting eggs)	Asexual (binary fission)
Generation time	3 months (sexual)	1 week (asexual)	Typically <1 day
Dispersal mode	Wind‐dispersed pollen	Swimming, currents, via resting eggs (e.g. on bird feet or via wind)	Water cycle, wind, rain, insect vectors
Offseason survival	Seed bank	Resting egg bank	Dormancy in soil or within tree host
Pathogen			
Size (longest axis)	30 μm (transmission spore)	35–60 μm	30–200 nm
Reproduction	Asexual transmission spores, possibly sexual resting spores	Parasexual	Asexual virions
Generation time	7–12 days (asexual)	10–20 days	Typically < 1 h
Transmission	Environmental, via wind	Environmental, host ingests free‐living spores in water	Environmental (passive)
Propagule release	Spores shed from live leaf	Obligate killer, spores released from dead host	Obligate killer, virions released from lysed cell
Dispersal range	1 m	Unknown	Unknown
Offseason survival	Resting spores on dead leaves	Unknown, but likely in sediment	Unknown, but possibly within bacterial genome
Host × pathogen			
Genetic specificity	Highly specific (gene for gene): recognition of pathogen avirulence allele by host resistance allele triggers defence responses. Also quantitative resistance	Genetic variation in host rate of parasite encounter and susceptibility given encounter, but no genetic variation in pathogen infectivity	Many known mechanisms of resistance/infectivity that vary from general to specific; local adaptation and infection network analyses often suggest high level of specificity
Environment			
Habitat	Dry meadows in Åland archipelago, Finland	Lakes in temperate North America	Horse chestnut trees in the United Kingdom
Growing season	July–September	July–November	May–September
Abiotic factors	Temperature, rainfall, humidity, wind	Temperature, light, UV and nutrients	Temperature, rainfall, nutrient availability
Biotic factors	Hyperparasites	Resources, predators and diluter species	Bacterial competition, tree defences

At the scale of individual hosts, the risk of infection and consequences of disease vary among hosts and across an individual's lifetime due to spatiotemporal variation in host traits (e.g. resistance and tolerance), parasite traits (e.g. infectivity and virulence) and the environment (e.g. microclimate and resource availability) (Fig. 1A). Within‐host parasite dynamics, including interactions between coinfecting strains, play a central role in determining the outcome of infection for host individuals (Koskella et al. 2006; Susi et al. 2015a). Indeed, an individual host can be thought of as an ecosystem in which parasites, commensals and immune components interact and compete for resources (Rynkiewicz et al. 2015). The spatial distribution of uninfected and infected hosts in a population varies greatly among systems depending on factors including habitat patchiness, host and parasite dispersal ability, and parasite transmission mode. For example, the herbaceous plant *Plantago lanceolata* grows patchily within meadows due to habitat constraints, and individuals infected with the powdery mildew *Podosphaera plantaginis* are further aggregated due to factors including a limited range of parasite dispersal and small‐scale genetic structure of the host and parasite (Laine 2006; Tack et al. 2014). Similarly, the interactions between culturable bacterial species and lytic bacteriophages from the phyllosphere of the horse chestnut tree, *Aesculus hippocastanum*, are shaped by the individual host tree in which they occur (Koskella et al. 2011), and there is good evidence that bacterial distribution within the phyllosphere is highly patchy, even within leaves (Esser et al. 2014). On the other hand, no within‐population spatial structure has been found for the zooplankton *Daphnia dentifera* infected by fungal spores of *Metschnikowia bicuspidata*, which hosts encounter in the water column of lakes (Hall et al. 2005). Specifically, Hall et al. (2005) found no gradient of infection prevalence with lake depth and little aggregation of infection on a horizontal scale of tens of metres, possibly because physical mixing mechanisms disperse fungal parasite spores within the lakes and disrupt biologically driven spatial patterning.

Box 1 **Women in science – our perspectives**

Upon being asked to contribute to this special issue on ‘Women's contribution to basic and applied evolutionary biology’, we sought to address a topical issue in the field with direct relevance to each of our own research programmes. The collaboration was easy and natural, with both expected but also surprising complementarity among our ideas, and we each learned a great deal from the process of writing this manuscript. Here we move beyond the science to each briefly outline a few key aspects of our experiences as women in this field.

**The role of advisors/mentors in shaping each of our careers**

*RMP*: A series of supportive advisors have nurtured my academic career thus far: Deane Mosher, in whose laboratory I began working as a high school student, my undergraduate advisor, Stephen Carpenter, PhD advisor, Meghan Duffy, and current postdoctoral advisor, Anna‐Liisa Laine. Working with mentors who were at very different stages in their own careers has given me valuable perspective on the academic career path. For example, as Meghan Duffy's first PhD student, I learned a lot about the early academic career stage and how to build a productive research group. Notably, my two most recent advisors are also wonderful role models as successful women in science. *A‐LL*: Growing up with a scientist for a mother has provided me with an example of a woman who is creative and passionate about her work. I worked mostly on my own during my PhD, but it was a good experience in every aspect. During that time, I was very much influenced by the works of Janis Antonovics, John Thompson, Jeremy Burdon and Peter Thrall, and I was lucky to carry out postdoctoral research with all of them before beginning my own group. *BK*: I have found the keys to success thus far have been: loving what I do; having mentors, advisors and advocates who support me, push me and speak up for me when need be; and surrounding myself with collaborators and students who love science as much as I do. My undergraduate advisor, Janis Antonovics, PhD advisors, Curt Lively and Mike Lynch, and postdoc advisors, Angus Buckling and John Thompson, always treated me as a scientific equal with great potential. This support went a long way in helping me fight the ‘imposter syndrome’ which still holds me back from time to time.

**The role of networking in building a scientific profile**

*RMP*: In addition to presenting at conferences, participating in workshops and using Twitter, moving abroad for postdoctoral research has helped me greatly expand my global network of scientists. I find interacting and collaborating with researchers from around the world on a daily basis to be invigorating and productive. *A‐LL*: Having met people from across the world with whom I share scientific interests has been the basis of many fun, productive collaborations. Discussion with peers, whether live or on Twitter, is an endless source of education and inspiration for me. Friendships with women scientists, with whom I have had open and lively discussion about pretty much everything, but also on being a woman in science, are an invaluable source of peer support. *BK*: Two avenues have really helped me to share my interest in science with a wider audience. First, I have been lucky to be invited to speak at a number of conferences, workshops and meetings since finishing my PhD. The financial support and accolade of the invitation has allowed me to build a wide international network. Second, my scientific network has been greatly expanded through Twitter and I have ‘met’ a number of international researchers I may never have interacted with otherwise. Finally, organizing and participating in discussions/panels focused on women in science has greatly expanded my network of female scientists and has helped me identify a number of excellent role models.

**The impact of pregnancy/motherhood on career progression**

*RMP*: I worked on this manuscript as a postdoc on maternity leave. Thanks to my supportive colleagues and baby's easy temperament, I often bring the baby to meetings and seminars. Having several months of paid maternity leave has allowed me flexibility to work according to my own unpredictable day‐to‐day schedule, with plenty of time to bond with my child. *A‐LL*: I truly believe that my two children make me a better scientist. Motherhood has helped me recognize my priorities and manage my time. Also, there is no better way of decompressing after work than being with kids, as they demand 100% of your attention. *BK*: I also worked on this manuscript while on maternity leave, which allowed for increased focus and efficiency. I am thankful to great and supportive colleagues and to those who use social media and blogs to share advice on balancing work and life. I look forward to putting much of this advice into practice moving forward as a mother in science.

At the scale of populations, the prevalence of a given parasite (i.e. proportion of hosts infected) typically varies dramatically over both space and time. This variation may reflect genetic differentiation across populations or over time, as well as spatiotemporal variation in community‐level interactions or abiotic conditions, which are frequently found to modulate the interaction between a given host and parasite genotype (Wolinska and King 2009). For example, peak prevalence of *M. bicuspidata* in populations of *D. dentifera* varies from 0% to more than 60% infected hosts among lakes in the Midwestern USA, with most epidemics peaking at <10% infected (Duffy et al. 2010; Hall et al. 2011b). For this and several other parasites of *D. dentifera*, among‐lake variation in peak infection prevalence was found to exceed that variation observed between years (Duffy et al. 2010). This result likely reflects both the lack of parasite dispersal between lakes and the fact that lakes vary strongly in habitat characteristics and ecological drivers of disease (Penczykowski et al. 2014). Among populations of *Pl. lanceolata* in the Åland Island of Finland, peak prevalence of *Po. plantaginis* ranges from 0% to more than 50%, but the majority of infected populations have <10% infection prevalence (A.‐L. Laine, unpublished data). In contrast to the *Daphnia* example above, high extinction and colonization rates of *Po. plantaginis* result in substantial fluctuations in prevalence among years, with the parasite persisting in most populations for only 1–2 years at a time (Jousimo et al. 2014). In the case of bacteria and phages from horse chestnut trees, susceptibility to infection has been found to vary spatially across trees, ranging from <10% to nearly 40% of host isolates, as well as between the surface and interior of leaves (Koskella et al. 2011), and has also been shown to vary dramatically across the growing season (Koskella 2013), with an average peak susceptibility of 35% occurring in July. Just as with disease prevalence, the consequences of disease for host populations are known to vary in space and time. For example, powdery mildew may cause greater mortality to *Pl. lanceolata* during times of drought (Laine 2004), and this type of environmental dependency may explain why host density was found to decrease following infection in some years more than others (Penczykowski et al. 2015). In the *D. dentifera–M. bicuspidata* system, epidemics that start earlier in the season and achieve greater maximum prevalence more strongly depress host densities than do smaller epidemics (Hall et al. 2011b); thus, spatiotemporal factors influencing infection peak prevalence drive ecological as well as evolutionary changes (Duffy et al. 2012).

At larger spatial and temporal scales, patterns of disease among populations can be studied to evaluate how host and parasite dispersal and environmental heterogeneity interact. Dispersal between populations can allow parasites to persist stably as a metapopulation despite ephemeral infection at the population level (e.g. in many plant pathosystems; Burdon and Thrall 2014). Dispersal ability and mode of reproduction will largely determine the degree of genetic structure of host and parasite populations across the metapopulation. For hosts or parasites with seasonal constraints, temporal genetic structure (e.g. whether there are genetic bottlenecks between seasons) may depend on their ability to store genetic information as seeds (e.g. for *Plantago* hosts) or diapausing stages (e.g. *Daphnia* resting eggs or powdery mildew resting spores; Table 1), or in the case of some parasites, to persist as free‐living stages or on alternate hosts. Large‐scale environmental heterogeneity may also determine which parasite populations establish, persist or go locally extinct. Because environmental factors are frequently spatially autocorrelated, the environment may also influence the degree of spatial synchrony in disease processes. For example, a shift towards milder winter conditions over a 13‐year time series in the Åland Islands likely eroded differences between populations in survival of the overwintering stage of *Po. plantaginis*, leading to increased spatial synchrony of disease occurrence across *Pl. lanceolata* populations in the region (Penczykowski et al. 2015).

The study of coevolution between hosts and parasites has also greatly benefited from examination over both temporal and spatial timescales. As the underlying genetics of host–parasite interactions are often difficult to uncover, much of our understanding of host–parasite coevolution comes from phenotypic measures of resistance and infectivity across time or space (reviewed in Gandon et al. 2008). Although the study of adaptation across space can be examined for most systems, those systems with hosts having short generation times and the ability to reproduce clonally are particularly conducive to testing for temporal adaptation. In particular, if hosts and parasites can be resurrected from the past (e.g. from natural banks of seeds, eggs or spores, or from frozen material), then ‘time shift’ experiments can be performed in which hosts from one point in time are exposed to parasites from another (Gaba and Ebert 2009; Fig. 1D). Furthermore, the use of experimental coevolution between hosts and their parasites has offered important insight to the factors influencing the mode and tempo of the coevolutionary process (reviewed in Brockhurst and Koskella 2013). Specifically, much can be learned by analysing coevolutionary dynamics following experimental manipulation of either the spatial (e.g. Brockhurst et al. 2003) or temporal (e.g. Morgan and Buckling 2006) structure of host–parasite interactions.

## The spatial scale of host–parasite interactions

Among the ways in which host–parasite interactions are typically examined across space are population genetic studies and local adaptation experiments. The examination of population genetic structure of host populations relative to interacting parasite populations can offer important insight both to the rate of dispersal of each species and also to the divergence among populations, as shaped by environmental heterogeneity and/or coevolution. Where this approach has been used, there has often been a strong asymmetry uncovered, with parasite populations showing much reduced structuring relative to their host populations (Dybdahl and Lively 1996; Davies et al. 1999; Keeney et al. 2009), or conversely much stronger differentiation than corresponding host populations (Delmotte et al. 1999; McCoy et al. 2005). In other cases, no relationship has been found between the genetic structure of host populations and that of their parasites (Mulvey et al. 1991). Among the reasons for such asymmetries and variation among systems are that the genetic structure of host and parasite populations depends on life histories of the organisms (reviewed in Barrett et al. 2008), including whether the parasite has a complex life cycle (Prugnolle et al. 2005) or a broad or narrow host range (Johnson et al. 2002).

Given the importance of dispersal for generating additive genetic variation upon which selection can act, it has been predicted that the antagonist with greater dispersal capability should be ‘ahead’ in the coevolutionary arms race (Gandon 2002). Indeed, comparisons across host–parasite systems suggest that in those systems where parasites have greater dispersal capability than their hosts, parasites tend to be better adapted to their host populations (Greischar and Koskella 2007; Hoeksema and Forde 2008). Similarly, experimental manipulation of migration rate has been shown to influence the ability of parasites to adapt to their local host populations (Morgan et al. 2005). There is also evidence of host local adaptation in systems where hosts have consistently higher gene flow than their parasites, for example in the case of innate immunity of pipe fishes against their local bacterial parasites (Roth et al. 2012). Such asymmetry in adaptation can have important consequences at the population genetic level, for example by hindering selective sweeps of host resistance alleles (Wilfert and Jiggins 2013). Overall, the population genetic structure of host and parasite populations both shapes and is shaped by migration across an often complex landscape. As such, an understanding of genetic structure across space can be very helpful in building predictions for disease emergence and spread.

### Local adaptation experiments

A common tool for studying the spatial scale of interactions among parasites and their hosts is the use of ‘local adaptation’ studies to compare the fitness of one antagonist when interacting with its local (or sympatric) population of the other antagonist relative to its fitness when interacting with foreign (or allopatric) populations (Blanquart et al. 2013; Fig. 1E). This measure offers insight into the coevolutionary process, as it can be used to examine divergence among populations for traits of interest to the interaction, but is not necessarily indicative of coevolution. For example, a pattern of parasite local adaptation (whereby sympatric combinations of hosts and parasites are more likely to result in successful infection than allopatric combinations) could simply reflect a parasite that is well adapted to host populations that are otherwise divergent across space; that is, that have diverged in the absence of parasite‐mediated selection. It does not, on its own, suggest that parasite‐mediated selection is playing a role in shaping the divergence among host populations. Furthermore, in the case of one‐sided host adaptation, it could be that host populations respond to local parasite‐mediated selection and are therefore well adapted, but that corresponding parasite populations are adapting primarily to a different host species. Evidence for host local adaptation against a generalist parasite has, for example, been documented in populations of *Arabidopsis thaliana* plant hosts tested against local versus foreign isolates of the generalist pathogenic bacterium, *Pseudomonas syringae* (Kniskern et al. 2011). Indeed, a systematic review across 32 local adaptation experiments demonstrated that generalist parasites were less likely to show a pattern of adaptation to local host populations than were specialist parasites (Lajeunesse and Forbes 2002). Moreover, when nonreciprocal measures of host and parasite fitness such as parasite infectivity and host tolerance are considered (i.e. as opposed to using infectivity/resistance as the fitness measure for both antagonists), it is possible for both players to show local (mal)adaptation simultaneously. For example, hen flea reproductive success was found to be lower on local versus foreign great tit hosts (indicating host local adaptation), and host fledglings were found to be smaller when infected with local relative to foreign fleas (indicating parasite local adaptation; Lemoine et al. 2012).

An obvious but critical starting point for designing any local adaptation study is the decision regarding the spatial scale at which comparisons are to be made. This decision can be informed by disease occurrence patterns, population genetic studies, physical barriers believed to reduce gene flow or known heterogeneity in other selection pressures acting across the landscape. The scale of local adaptation can vary greatly across systems, even for those that have similar life histories such as fungal plant parasites, which have been observed to be locally adapted at the level of the individual host plant (Capelle and Neema 2005), the population and metapopulation levels (Laine 2005), and at the regional scale (Thrall et al. 2002). Alternatively, local adaptation can be measured across multiple spatial scales simultaneously to identify the range that is most meaningful for study of a given interaction (Imhoof and Schmid‐Hempel 1998; Thrall et al. 2002; Laine 2005). For example, in order to determine the spatial scale of phage adaptation to populations of bacterial hosts from horse chestnut leaves, cross‐inoculations were run between phages and bacteria collected either from different leaves within the same tree host or from across different tree hosts (Koskella et al. 2011). In this case, phages were found to be locally adapted to bacteria collected from the same tree relative to bacteria from neighbouring trees, regardless of how far apart they were spatially, but were no more or less infective to bacteria collected from other leaves within the same tree. This result suggests that the spatial scale of the bacteria–phage interaction in this system is meaningfully shaped by the biotic environment, rather than physical distance, a pattern in stark contrast to what had been previously observed for phages from the soil, where phages were found to be less infective to bacterial hosts from only centimetres away (Vos et al. 2009). A similar result was found for the *Linum marginale*–*Melampsora lini* plant–parasite system, as the parasite was found to be locally adapted across a regional scale, with no effect of geographic distance among populations observed (Thrall et al. 2002). Finally, just as local adaptation can vary across spatial scales examined, so too can it vary among populations across a heterogeneous landscape. For example, in the *Pl. lanceolata–Po. plantaginis* system, both the strength and direction of parasite local adaptation were found to differ among populations along a temperature gradient (Laine 2008). Similarly, phage populations that were experimentally coevolved with the bacterium *Pseudomonas fluorescens* were found to have a stronger signature of local adaptation when tested against allopatric populations that differed in their nutrient concentration than allopatric populations with similar nutrient levels (Lopez‐Pascua et al. 2012).

### Spatial scales of host resistance

The spatial distribution of host resistance is expected to fundamentally affect epidemiology, as we can only find disease when host defence strategies are overcome. Host resistance may also be considered the main driving force of parasite evolution, with parasites evolving to escape local host resistance strategies. There is remarkably little direct evidence from natural populations for hosts evolving resistance under parasite attack (but see examples discussed below), although variation in disease resistance is widespread (Salvaudon et al. 2008; Laine et al. 2011). This implies that natural host populations have the capacity to undergo significant adaptive evolution in response to parasite attack. How much hosts invest in resistance needs to be balanced along the axes of how costly resistance (Bergelson and Purrington 1996) versus infection (Susi and Laine 2015) are to the host in terms of impacting fitness, and on the resource availability to the host (Lopez‐Pascua and Buckling 2008; Hall et al. 2010; Lopez‐Pascua et al. 2014). These factors may vary through space and time, for example being influenced by the biotic (Koskella et al. 2012) and/or abiotic (Auld et al. 2013) environment, generating variation in how hosts evolve resistance. Hence, examining how disease resistance is spread across space can offer otherwise difficult to attain insights into the processes that drive host–parasite interactions given the challenges of directly documenting coevolution (Gaba and Ebert 2009). A recent review of plant–parasite interactions confirmed that variation in resistance, as measured in controlled inoculation trials, is ubiquitous across all scales examined ranging from molecules to metapopulations (Laine et al. 2011). Despite this variation, susceptibility is more common than resistance, a phenomenon best explained by fitness costs of resistance to the host and the ability of parasites to rapidly adapt to novel resistances (Laine et al. 2011). Another possibility is that the result reflects a bias in the systems and/or populations chosen for host–parasite studies, as they will likely be chosen initially based on the presence of disease. Indeed, susceptibility to one parasite is easier to measure than resistance to all other possible parasites that are not observed on the host.

An ideal system for studying evolution of host resistance would have no heritable genetic variation in parasite infectivity – that is, no possibility for coevolution. One such system is the interaction between *D. dentifera* and *M. bicuspidata* (Table 1). *Metschnikowia bicuspidata* traits can respond plastically to different host environments (Searle et al. 2015), but there is no variation in infectivity or virulence among isolates collected from different lakes (Duffy and Sivars‐Becker 2007; Searle et al. 2015). The parasite also has not responded to selection in laboratory experiments (Duffy and Sivars‐Becker 2007; Auld et al. 2014). This lack of heritable variation in parasite traits provides an opportunity to test directly for parasite‐driven evolution of host traits. Moreover, because the size of epidemics and strength of selection varies among lakes, this system has been used to assess patterns and drivers of spatial variation in evolution of host resistance. Ecological drivers of disease, including resource availability and predation pressure, modulate the size of epidemics and strength of selection for resistance versus fecundity; thus, spatiotemporal variation in these ecological factors can lead to divergent evolutionary outcomes across populations or time (Duffy et al. 2012). Indeed, within epidemic seasons, evolution of increased resistance, increased susceptibility, disruptive selection on resistance or no change in resistance level have all been documented across *D. dentifera* populations (Duffy and Sivars‐Becker 2007; Duffy et al. 2008, 2012). Erosion of genetic variation for resistance during a given epidemic may in turn determine the slope of the resistance trade‐off and potential for host evolution during subsequent epidemics (Auld et al. 2013).

A high level of diversity in resistance phenotypes has been shown to protect host populations against parasites, and variation among resistance loci within host individuals constitutes a fundamental component of this diversity (Laine et al. 2011). In *Pl. lanceolata,* the same host genotype is typically resistant to some strains of *Po. plantaginis* while being susceptible to others (Laine 2004, 2006), leading to pronounced variability among host individuals within populations. Within‐population diversity ranged from every individual representing a unique resistance phenotype to half of the individuals sharing the same phenotype (Laine 2004). Resistance was higher in areas within host populations where disease encounter rates have been systematically high than in areas where they have been low, providing one of the few examples of divergent parasite selection within host populations (Laine 2006). The fine‐scale selection mosaic may have formed through an interaction with the physical environment, as the study coincided with severe drought with the highest levels of mortality in areas of the populations where disease had been most prevalent (Laine 2006).

When variation in resistance is examined among populations, some studies find significant differences in the average level of resistance observed (Thrall et al. 2002; Niemi et al. 2006), while other systems show relatively similar overall levels of resistance (Carlsson‐Granér 1997; Thrall et al. 2001). In the *Pl. lanceolata*–*Po. plantaginis* interaction, populations with a history of infection have more similar levels of resistance than those host populations that were known to be uninfected for several successive years (Laine 2004, 2005). Interestingly, and possibly because of variation in disease history even among neighbouring host populations (Jousimo et al. 2014), no evidence was found for greater similarity in the resistance phenotypic compositions of neighbouring than far‐away populations (Laine 2004). An analysis of 13 years of epidemiological data in this system revealed that the parasite was less likely to establish or persist in highly connected host populations, suggesting that population level resistance is higher in dense host networks than in isolated host populations. This hypothesis was confirmed by a laboratory inoculation study (Jousimo et al. 2014). Jointly, these results demonstrate how landscape configuration may generate variation in evolutionary trajectories among populations, in addition to divergence driven by variation in the abiotic and biotic environment (Wolinska and King 2009; Laine et al. 2014).

Viewing interactions across multiple populations within a metapopulation demonstrates that short‐term changes within populations may differ from the evolutionary trajectory of the entire metapopulation (Thrall and Antonovics 1995; Smith et al. 2011). At an even larger spatial scale, metapopulations may have different evolutionary trajectories (cf. Thompson 2005). However, to date, there are few data available on the resistance structure of wild host populations that cover large regional spatial scales. In the interaction between wild flax and its rust disease in Australia, genetic and phenotypic structure of resistance is markedly different among regions that differ in their environmental conditions, life histories and mating systems. The region with outcrossing hosts showed greater diversity of resistance and infectivity phenotypes, higher levels of resistance and less clumped within‐population spatial distribution of resistance (Nemri et al. 2012).

### Spatial scales of parasite infectivity and virulence

Variation in parasite infectivity and virulence, and how this variation is spatially structured, is important to quantify as it provides the raw material for antagonistic (co)evolution and therefore underlies risks of disease spread and host shifts. Moreover, examining spatial variation in these parasite traits may inform us about the underlying processes driving the evolution of parasite populations (e.g. Osnas et al. 2015). A recent review demonstrated that variation in pathogenicity is pervasive across multiple spatial and temporal scales (Tack et al. 2012). Variation in infectivity among parasite isolates was omnipresent, as each study system contained multiple parasite strains that varied in their ability to infect different host plant genotypes. In general, the magnitude of within‐population variation in pathogenicity is large relative to among population variation, and the distribution in this variation partly mirrors the distribution of host resistance (Tack et al. 2012). In the metapopulation of *Po. plantaginis*, approximately half of the local parasite populations consist of a single strain, while half support several parasite strains that typically vary in their infectivity and virulence (Tollenaere et al. 2012).

Variation in infectivity and virulence among parasite populations is frequently found (Tack et al. 2012; Osnas et al. 2015) and much of this variation is adaptive, suggesting coevolution with resistance of the host is a major driver of parasite variation across space (Thrall et al. 2002; Laine 2005; Greischar and Koskella 2007; Koskella 2014). In the interaction between *Pl. lanceolata* and *Po. plantaginis*, the parasite was found to be locally adapted at the scale of clusters of host populations rather than individual host populations, demonstrating how intertwined spatial population processes and evolutionary dynamics are. Environmental variation may also generate among population variation in parasites. In the interaction between *L. marginale* and *M. lini*, host ecotypes growing in different habitats yet in close proximity also selected for among population variation in the parasite (Laine et al. 2014). Much of this among habitat variation is thought to be maintained by differences in soil moisture and microbiota (Tack et al. 2015). Given that variation among parasite populations within metapopulations is omnipresent, it is not surprising that variation in pathogenicity is also universal at larger spatial scales (Tack et al. 2012).

### Linking within and between host dynamics

The issue of multiple infections, whereby more than one strain of the same or different parasite species simultaneously infect the same host individual, has been a topic of considerable interest for studies of human and animal–parasite interactions. The interest stems from the notion that within‐host dynamics among parasite strains is expected to have consequences for host–parasite dynamics, with predicted effects on the evolution of virulence and transmission ability (Alizon et al. 2013). Although coinfection is now considered a widespread phenomenon, and experimentally it has been shown to change infection outcomes, little is currently known about how coinfection may alter disease dynamics during epidemics. Common garden populations of *Pl. lanceolata* infected either singly or coinfected by two *Po. plantaginis* strains showed that disease dynamics change under coinfection resulting in higher disease prevalence at both genotype and population levels (Susi et al. 2015a). This change was best explained by higher transmission from coinfected than from singly infected hosts (Susi et al. 2015a,b). These experimental findings were confirmed in natural parasite populations—more devastating epidemics were measured in populations with higher levels of coinfection (Susi et al. 2015a). Jointly, these results confirm the predictions made by theoretical and experimental studies for the potential of coinfection to alter disease dynamics across a large host–parasite metapopulation (Susi et al. 2015a). The study by Susi et al. (2015a) also discovered higher levels of coinfection in highly connected parasite populations than in more isolated ones. Again, this highlights the importance of dispersal among populations for the evolutionary and epidemiological dynamics of parasites.

The presence of different strains infecting the same host increases the likelihood for the evolution and emergence of new variation in pathogenicity. For many parasites, coinfection is the prerequisite of sexual reproduction, and even among asexual parasites, coinfection promotes exchange of genetic information (Smillie et al. 2011). Furthermore, theory predicts that the existence of multiple infections may facilitate the maintenance of polymorphism within local populations via competitive interactions (Nowak and May 1994). Likewise, coexistence of parasites could be mediated by a trade‐off where more virulent parasites have less potential for dispersal due to a shorter lifetime of the host, but are better within‐host competitors (Anderson and May 1982). Finally, genetic diversity of the parasite population may also influence the frequency at which coinfections occur. López‐Villavicencio et al. (2007) observed that high genetic diversity within parasite populations may result in fewer coinfections, seemingly due to higher within‐host competitive exclusion among unrelated strains. Similarly, a study on the anther smut fungus, *Microbotryum violaceum*, demonstrated that coinfection was a more likely outcome for related strains than for less related strains (Koskella et al. 2006), which could have important consequences for the success of migrant parasites into heavily infected host populations. As such, the outcome of within‐host dynamics may well play a role in shaping the spatial structure of host–parasite interactions.

## The temporal scale of host–parasite interactions

As discussed above, the strength of and response to parasite‐mediated selection in nature typically varies across both space and time. This variation can be driven by selection mosaics across the landscape (Forde et al. 2004) or can result from temporal factors such as the seasonality of epidemics (Altizer et al. 2004). While there are a number of approaches to examine the spatial scale of host–parasite interactions, less emphasis has typically been placed on studying the temporal scale of the interaction. This is partly due to the difficulty in tracking dynamics over time and partly due to the paucity of systems for which the loci involved in infection/resistance have been identified. At a macroevolutionary scale, co‐phylogenies between hosts and parasites can be used to identify when given lineages of each species diverged and whether divergence is mirrored across the two phylogenies (e.g. Bellec et al. 2014). Such macroevolutionary analyses have been used to infer cospeciation and host shifts for some time, but cannot offer direct insight to the coevolutionary process or rate at which change is occurring within populations (de Vienne et al. 2013). On the other hand, experimental evolution studies have been used to highlight the great speed at which host populations can respond to parasite‐mediated selection and vice versa, but do not provide direct evidence that coevolution is happening or will happen in natural populations. In the laboratory, a response to parasite‐mediated selection can occur extremely rapidly when selection is acting on standing variation [over the course of six generations for snails and their trematode parasites (Koskella and Lively 2009), <20 generations for *D. dentifera* during *M. bicuspidata* epidemics (Duffy and Sivars‐Becker 2007), and 48 generations for a nematode host coevolving with a bacterial parasite (Schulte et al. 2010)], but can also occur rapidly when experiments are initiated with a single host and parasite clone (ongoing coevolution between phages and their bacterial hosts has been demonstrated over the course of 15 bacterial host generations; Buckling and Rainey 2002). The speed of host–parasite coevolution in nature will of course be shaped by the strength of selection and the additive genetic variation within populations (in the form of standing variation, migration or mutation), both of which are likely to differ across space, thus creating so‐called hot spots and cold spots of coevolution (Thompson 2005). The response to selection will also be influenced by the myriad of other selection pressures acting on the populations, for example those resulting from other parasites, predators or competitor species, and these are typically absent in laboratory experiments. Recent evidence from natural populations of the freshwater snail, *Potamopyrgus antipodarum*, that differed in parasite prevalence over the course of 5 years, suggested more rapid change in the clonal composition of hosts within populations facing strong parasite‐mediated selection than in those under more relaxed selection (Paczesniak et al. 2014). Importantly, the pace of coevolution in one population can have a significant impact on other populations connected by gene flow. For example, manipulation of the encounter rate between the bacterium, *Pseudomonas fluorescens,* and its phage, SBW25Φ2, demonstrated that the speed of experimental coevolution within populations linked by one‐way migration was affected by the source population from which migrants were arriving (Vogwill et al. 2009). Populations with increased encounter rate, and therefore which were ‘hotspots’ of coevolution, acted to speed up the rate of coevolution in connected ‘cold spot’ populations, while migrants from ‘cold spot’ populations acted to slow down the rate of coevolution within connected ‘hot spots’.

### Time shift experiments

One very powerful tool for measuring the rate of coevolutionary change is the use of time shift experiments (reviewed in Gaba and Ebert 2009; Brockhurst and Koskella 2013), whereby the antagonist from one point in time is challenged against the other antagonist from either past, contemporary or future points in time (Fig. 1D). In its most basic form, the host–parasite time shift experiment allows a researcher to ask whether the fitness of populations from the future is higher than the fitness of the contemporary and/or past populations when tested against a fixed population of the antagonist, thus suggesting the focal population has responded to selection imposed by the antagonist over the time period examined. Similarly, the researcher can ask whether fitness is lowest for populations from the past, which have not yet responded to any adaptations of the contemporary antagonist. When such dynamics are observed for both antagonists, the results indicate a coevolutionary escalation, whereby each antagonist is responding to selection imposed by the other over the time points examined. For example, by freezing subsamples of bacteria and phage populations over the course of experimental coevolution in the laboratory, rapid coevolution was evidenced by highest bacterial resistance against ancestral phage and lowest bacterial resistance against phages from 15 bacterial generations in the future (Buckling and Rainey 2002). Such evidence is not restricted to laboratory model systems (Decaestecker et al. 2007); recent work applying the same time shift approach to natural populations/communities of bacteria and phages from the horse chestnut phyllosphere observed a similar pattern (Koskella 2013). In this case, bacteria were found to be, on average, most resistant to phages from a month earlier in the season and least resistant to phages from a month later in the season. A critical starting point for any time shift experiment is the choice of a temporal window across which to test for coevolution, and without prior information, it is possible to miss the appropriate time scale. For example, in a time shift experiment performed between water flea hosts, *Daphnia magna*, and the bacterial microparasite, *Pasteuria ramosa*, collected from various depths of a lake sediment core, peak fitness was observed for contemporary combinations of host and parasite, rather than past time points (Decaestecker et al. 2007). In this case, the authors put forward a theoretical model to support the possibility that the sediment layers used represented populations that were too temporally distant to accurately capture the rapid coevolutionary response occurring in nature. Therefore, when possible, it is best to perform times shifts across multiple time points spanning the predicted window over which coevolution is expected to occur.

Testing fitness across multiple past time points also allows for a powerful test of the underlying mode of coevolution (Gaba and Ebert 2009); differentiating between arms race dynamics, whereby parasite infectivity and host resistance increase directionally over time, and fluctuating selection dynamics, whereby host resistance and parasite infectivity are highest against antagonists from the recent past but less resistant/infective against antagonists from further in the past, is only possible using this approach. Evidence for fluctuating selection can be indicative of negative frequency‐dependent selection, whereby hosts/parasite populations adapt to common genotypes and, in doing so, lose the ability to resist/infect previously common genotypes, or suggestive of fitness trade‐offs for resistance/infectivity. Fluctuating selection dynamics have been observed in the horse chestnut phyllosphere, where phages were found to be most infective to bacterial hosts from the recent past, but were less likely to be infective to bacteria from 4 months earlier in the season (Koskella 2014). This is in contrast to what is often observed for bacteria and phages coevolving in the laboratory, where time shift experiments more often uncover arms race dynamics (e.g. Buckling and Rainey 2002; Gandon et al. 2008). The reason for this difference may be elucidated by the incorporation of more ecologically relevant parameters into experimental coevolution studies. Evidence from the *P. fluorescens–*phage SBW25Φ2 system suggests that even in the laboratory, longer‐term experimental evolution shows evidence for decelerating arms race dynamics (Hall et al. 2011a), and the incorporation of other competitor bacterial species in the microcosm environment has also been shown to move the dynamics of this system away from arms race dynamics and towards fluctuating selection dynamics (Gómez and Buckling 2011). Finally, it is important to note that ruling out fluctuating selection dynamics when interpreting time shift results is complicated by the possibility that the window of time in which the interaction was tested does not go backwards (or forwards) in time sufficiently far to see the loss of parasite infectivity/host resistance (Gaba and Ebert 2009). However, this problem is easily circumvented when comparing the results across multiple treatments (in the case of experimental coevolution) or populations. For example, recent work using time shift experiments to examine coevolution between the bacterium *Pseudomonas aeruginosa* and a number of different lytic phages in the laboratory demonstrates that different pairwise interactions can result in different patterns of coevolution across the same absolute timescales, although this pattern could be explained in part by different relative timescales if the phage generation times differed (Betts et al. 2014). Furthermore, comparison across experimental microcosms with high versus low nutrient availability suggests that increased productivity of host populations accelerates the rate of coevolution (Lopez‐Pascua and Buckling 2008), while also shifting dynamics away from fluctuating selection dynamics and towards arms race dynamics (Lopez‐Pascua et al. 2014). Data such as these highlight that the tempo and mode of coevolution can vary substantially across otherwise similar systems as a function of the abiotic and biotic environment, as well as depending on the infection genetics underlying the interaction.

### Eco‐evolutionary dynamics in host–parasite interactions

Ecological processes drive evolution through natural selection, and it is now recognized that evolution can also occur on an ecological timescale (Jones et al. 2009). As mentioned in the preceding section, parasite‐driven evolution of hosts, and vice versa, can occur over the course of a few generations. Evolution of host and/or parasite traits can even occur within the course of an epidemic, such that evolutionary change shapes the outcome of the epidemic in terms of host and parasite densities (reviewed in Penczykowski et al. 2011). These ecological changes may in turn modulate the strength of selection and drive subsequent evolution in the pathosystem.

Our understanding of host–parasite interactions inherently recognizes the tight link between evolutionary and ecological dynamics. Disease dynamics are formed through eco‐evolutionary feedback loops in that parasites can only infect hosts whose resistances they have evolved to overcome. When we consider wild host–parasite interactions, an overwhelming number of studies have convincingly illustrated that coevolutionary interactions among hosts and parasites play a major role in explaining spatial and temporal variation in host and parasite traits, as well as local adaptation. While coevolution alone does not imply a link between evolutionary and ecological timescales, it is becoming increasingly clear that host–parasite coevolution and local adaptation can be rapid (e.g. Laine 2005, 2006; Thrall et al. 2012) and therefore have the potential to impact ecological dynamics.

Theory predicts that evolution of host defences and the resulting impact on host–parasite ecological dynamics will depend on traits of the host and parasite, the type of host defence (e.g. resistance or tolerance) and the costs of investing in that defence (Boots et al. 2009a). The potential for eco‐evolutionary dynamics in host–parasite systems has been addressed both experimentally and in the field. The importance of environmental context and spatial structure in eco‐evolutionary feedbacks has been demonstrated experimentally using the Indian meal moth *Plodia interpunctella* and its granulosis virus. In that system, greater host resistance evolved when selection occurred under higher resource conditions, despite similar infection risk under lower resources, suggesting that more resources allowed costs to be paid for host resistance (Boots 2011). In addition, experimental manipulation of dispersal rates showed that less dispersal (more local interactions) resulted in decreased parasite infectivity (i.e. weaker parasite‐mediated selection) (Boots and Mealor 2007) and had a clear influence on host population dynamics over time (Boots et al. 2009b). Similarly, experimental manipulation of migration rates of *Escherichia coli* bacteria and their T4 phages in a metapopulation was found to drive evolution of fast (with more migration) or slow (with less migration) rates of phage replication, which in turn drove ecological dynamics (Kerr et al. 2006). An example of eco‐evolutionary dynamics in nature comes from the *D. dentifera–M. bicuspidata* system, where theoretical models confirmed that observed within‐season evolution of increased host resistance may be a key driver of seasonal declines in infection prevalence (Duffy and Sivars‐Becker 2007; Duffy et al. 2009). Diseases of crops provide some of the most powerful examples of how the link from evolution to ecology may operate. In fact, the whole concept of resistance breeding in crops is based on the underlying assumption of a direct evolution‐to‐ecology pathway, so that a newly evolved resistance is expected to have a direct impact on epidemiology (Deadman 2006). The boom‐and‐bust dynamics in agricultural pathosystems provide the most convincing evidence for rapid evolution and its impact on spatial population dynamics. During the ‘boom’ phase, a resistant cultivar with a single major resistance gene is introduced into an agricultural system to reduce disease prevalence and is employed widely. However, the ‘boom’ phase is frequently followed by the ‘bust’ phase when an evolutionary change in the pathogen population breaks down host resistance (McDonald and Linde 2002; Deadman 2006). Consequently, the newly evolved pathotype rapidly spreads and infects all fields with the previously resistant cultivar. A current and pressing example of boom‐and‐bust dynamics is the Ug99 strain of stem rust (*Puccinia graminis*), which can overcome the resistance of most of the world's wheat varieties (Stokstad 2007). Appearing in Uganda in 1999, it has since spread into South Africa, Yemen and Iran and threatens wheat crops throughout the Middle East and West Asia (Singh et al. 2011).

## Combining spatial and temporal scales of host–parasite interactions

Recently, there has been a push to take a combined approach of examining host–parasite interactions across time and space simultaneously (Blanquart and Gandon 2013; Burdon and Thrall 2014; Koskella 2014). The examination of patterns of host/parasite adaptation across space has typically been considered a more rapid approach for inferring coevolutionary dynamics over time (Gandon et al. 2008; Burdon and Thrall 2014) and/or been used to examine differing patterns of coevolution across spatial heterogeneous landscapes (Thompson 2005). But more recently, there has been specific interest in examining how patterns of adaptation across space might differ over time. Experimental coevolution of *Escherichia coli* and the bacteriophage T7 within microcosms that were connected via varying degrees of gene flow and differed in productivity was used to demonstrate that patterns of phage adaptation can change both over time and across an abiotic gradient (Forde et al. 2004). Importantly, although local adaptation studies across space have been used to draw conclusions regarding which antagonist is ‘ahead’ in the arms race, there need not be a correlation between signatures of local adaptation across space and the rate of adaptation across time. For example, examination of bacteria and phages coevolving in soil microcosms demonstrates that while phages tended to be locally adapted across space (such that they were more infective to bacterial hosts from sympatric microcosms), it was the bacterial host that showed a greater rate of adaptation when examined temporally via a time shift experiment (Gómez and Buckling 2011). As such, the combination of time shift experiments and local adaptation experiments can be used to directly compare ‘temporal adaptation’ and ‘local adaptation’ in order to determine whether adaptation is more pronounced over space or time, and indeed whether such patterns are reciprocal for host and parasite. A comparison of adaptation of HIV viral populations against human antibodies from either the same or different hosts (local adaptation) or from past, contemporary or future time points within the same host (temporal adaptation) was recently used to illustrate the power of this technique (Blanquart and Gandon 2013). By decomposing viral fitness, the authors were able to demonstrate both changing immunity over time and viral evolution, despite a strong signature of local maladaptation across individual hosts within contemporary time. This again emphasizes that the translation of spatial local adaptation data into inferences about coevolutionary dynamics over time must be made with caution.

Another useful approach combining measures of adaptation across time and space is the use of time shift experiments across past, contemporary and future time points from both sympatric and allopatric combinations simultaneously (Koskella 2014). This approach allows for direct examination of temporal adaptation, but also addresses the specificity of such adaptation. Time shift experiments run only within sympatric combinations can uncover patterns of increased fitness against antagonists from the recent past (in the case of fluctuating selection dynamics) or escalating fitness against all past antagonist populations (in the case of arms race dynamics), but these patterns do not allow for examination of the specificity of such adaptations. In other words, it could be that parasites become generally more infective over time or it could be that the peak fitness observed in past time points is highly specific to the sympatric host population. For phages adapting to bacterial hosts in the horse chestnut phyllosphere, this approach was recently used to uncover a more pronounced pattern of phage local adaptation when measured on bacterial hosts from the recent past (Koskella 2014). In this case, phages were found to be most infective to their bacterial populations from a month or two earlier in the season, and this peak in fitness was highly specific to the sympatric populations. The subsequent decrease in infectivity against hosts from 4 months earlier in the season was indicative of fluctuating selection dynamics, but also meant that the signature of local adaptation was decreased when tested at these earlier time‐shifted points. Thus, the observed pattern of phage adaptation in this system was found to be specific over both space and time. Expanding this combined approach to other systems will provide important information regarding the specificity of temporal adaptation of hosts and parasites, and may also be useful in uncovering patterns of host local adaptation against parasites from the recent past (which have not yet responded to any changes in host resistance), even for systems in which parasite local adaptation across space is the norm.

## Conclusions/future directions

Studies of host–parasite interactions in nature, including phenotypic, genotypic and epidemiological observations across spatial/temporal scales, local adaptation experiments and time shift experiments, can reveal important insights into host–parasite coevolution and its ecological consequences. Such studies are complemented by experimental coevolution, which allows for direct tests of mechanisms and consequences of coevolutionary dynamics. A key result emerging from the body of literature reviewed here is that environmental heterogeneity at each spatial and temporal scale can strongly shape host–parasite interactions and the mode of coevolution. This environmental heterogeneity includes variation in both abiotic and biotic factors, ranging from within‐host competition among parasite strains or fluctuations in within‐host resources to environmental and ecological variation among metapopulations. Indeed, a general consensus is that expression of host resistance (or parasite infectivity) is far from the stable phenotype assumed in many epidemiological models and models of host–parasite coevolution. Thus, the more data we acquire on the spatial and temporal variation in natural host–parasite interactions as well as the consequences of such variation under controlled laboratory settings, the more accurately we will be able to translate evolutionary and ecological theory into predictions for the emergence, spread and dynamics of disease.

The three case studies highlighted in this review illustrate that differences in biological and environmental features among host–parasite systems (Table 1) necessitate different spatial and temporal scales of analysis to understand their (co)evolutionary dynamics. Determining the appropriate scales of analysis is critically important, as experiments or sampling schemes of too short duration or spatial scope, or with too large of temporal or spatial separation between observations, may miss key coevolutionary dynamics. We suggest that, when possible, the combination of local adaptation and time shift studies can offer new insight to the specificity of coevolutionary dynamics and can further untangle asymmetries in host and parasite adaptation. This approach has uncovered highly specific adaptation of phages to their bacterial hosts from horse chestnut trees, with temporal specificity of a few months and spatial specificity of trees 25–450 m apart (Koskella 2014). Future experimental work testing the interaction of *Pl. lanceolata* and *Po. plantaginis* collected from different years across a large metapopulation may yield much needed insight into the temporal scale of local adaptation in this plant pathosystem. Even in the *D. dentifera–M. bicuspidata* system, where the parasite is not evolving in response to its host, studies across lakes and years reveal evolutionary dynamics of host resistance that would be missed by studies of a single population or epidemic (Duffy and Sivars‐Becker 2007; Duffy et al. 2008, 2012; Auld et al. 2013). Hence, a general conclusion from these focal systems is that a spatiotemporal approach is key to answering central questions in disease biology: When, where and how should we expect hosts and parasites to evolve in response to each other?

The study of host–parasite interactions across scales is increasingly important as there is building evidence that these scales are changing due to human‐mediated factors including climate change, habitat fragmentation (Opdam and Wascher 2004) and increased dispersal (Altizer et al. 2013; Alexander et al. 2014). Importantly, these and other types of anthropogenic change may lead to spatial or temporal mismatches between antagonists, resulting in host shifts, altered parasite virulence and the rapid spread of disease across susceptible host populations not historically exposed to particular parasites. A recent review of fungal pathogens has emphasized the immediacy of this question, describing the impact of farming, landscape change, trade/movement and climate fluctuations on the rapid emergence and spread of new fungal pathogens threatening animal and plant species alike (Fisher et al. 2012). We have argued that the simultaneous examination across multiple scales and/or integration of data from multiple scales for model systems can provide novel insight that would not be possible to attain from data collected at a single scale. However, as of yet there are few systems for which such broad data sets are available and we are only beginning to reach the full potential of such combined approaches. Recent advances in sequencing technologies, bioinformatical analyses, as well as the continued collection of long‐term data sets, mean that the field is now in a unique position to begin addressing these key questions in disease ecology and evolution across scales. For example, new statistical tools for examination of interaction networks allow for analysis of data at the level of entire ecological communities (Toju et al. 2014) and can be used to demonstrate the impact of shared parasites on the evolution of multiple host species (Pilosof et al. 2014). Similarly, the introduction of single cell sequencing can be used to more accurately study host*–*parasite interactions (Labonté et al. 2015) as well as within‐host dynamics of multiple strains or parasites (Nair et al. 2014). This type of data may be particularly useful when examined within the theoretical framework developed for community ecology, as advocated by Seabloom et al. (2015). Together, these new combined approaches and reanalyses of older or long‐term data sets hold great potential to advance our understanding of host–parasite interactions and increase our ability to control and manage disease in natural, agricultural and human populations.
